# TeraStitcher - A tool for fast automatic 3D-stitching of teravoxel-sized microscopy images

**DOI:** 10.1186/1471-2105-13-316

**Published:** 2012-11-27

**Authors:** Alessandro Bria, Giulio Iannello

**Affiliations:** 1 Center of Integrated Research, University Campus Bio-Medico di Roma, v. Alvaro del Portillo 21, 00128 Roma (RM), Italy; 2 Department of Electrical and Information Engineering, University of Cassino and L.M., v. Di Biasio 43, 03043 Cassino (FR), Italy

**Keywords:** Stitching, 3D images, Teravoxel-sized images, Microscopy, Brain imaging

## Abstract

**Background:**

Further advances in modern microscopy are leading to teravoxel-sized tiled 3D images at high resolution, thus increasing the dimension of the stitching problem of at least two orders of magnitude. The existing software solutions do not seem adequate to address the additional requirements arising from these datasets, such as the minimization of memory usage and the need to process just a small portion of data.

**Results:**

We propose a free and fully automated 3D Stitching tool designed to match the special requirements coming out of teravoxel-sized tiled microscopy images that is able to stitch them in a reasonable time even on workstations with limited resources. The tool was tested on teravoxel-sized whole mouse brain images with micrometer resolution and it was also compared with the state-of-the-art stitching tools on megavoxel-sized publicy available datasets. This comparison confirmed that the solutions we adopted are suited for stitching very large images and also perform well on datasets with different characteristics. Indeed, some of the algorithms embedded in other stitching tools could be easily integrated in our framework if they turned out to be more effective on other classes of images. To this purpose, we designed a software architecture which separates the strategies that use efficiently memory resources from the algorithms which may depend on the characteristics of the acquired images.

**Conclusions:**

TeraStitcher is a free tool that enables the stitching of Teravoxel-sized tiled microscopy images even on workstations with relatively limited resources of memory (<8 GB) and processing power. It exploits the knowledge of approximate tile positions and uses ad-hoc strategies and algorithms designed for such very large datasets. The produced images can be saved into a multiresolution representation to be efficiently retrieved and processed. We provide TeraStitcher both as standalone application and as plugin of the free software Vaa3D.

## Background

### Advances in microscopy technologies

The recent advances in modern microscopy enable three-dimensional imaging of large biological specimens at high resolution. Often, the sample is too large to fit into the field of view of the microscope, so that a combination of multiple overlapping recordings (*tiles*) is needed to enable the reconstruction (*stitching*) of the whole image from the individual image stacks. Software 3D-Stitching tools are needed to assemble tiles since in many cases reliable tile positions are not available. This is true also when motorized stages are used for fast and reproducible acquisition of multiple tiles, since their physical coordinates may not be precise enough to allow direct reconstruction.

Current microscopy technology can easily require image reconstructions starting from tens of tiles, each with sizes ranging from tens of megavoxels (e.g. 1024×1024×64) to hundreds of megavoxels (e.g. 2048×2048×128). Several stitching tools have been therefore recently developed to deal with such a challenging task, and successful stitching of 3D images of these orders of magnitude has been reported in the literature [1-3].

However, the growing demand to acquire complete and large biological specimens at high resolution has lead to further advances in microscopy. In particular, the class of microscopes based on the technique commonly referred to as ultramicroscopy or light sheet-based microscopy (LSM) [4] enables fast three-dimensional high resolution imaging of large specimens exploiting their induced fluorescence. Samples are illuminated by a thin sheet of light and the fluorescence emission is observed from the axis (scanning axis) perpendicular to the illumination plane. Objects are then translated along the scanning axis, so obtaining a tile as a stack of 2D slices [5]. Combining this technique with a special procedure to clear tissue makes it possible to acquire large specimens, such as whole mouse brains [6]. Recently, at LENS in Florence, it has been developed an improved LSM technique, referred to in the following as *Confocal Light Sheet Microscopy* (briefly CLSM), which is capable of imaging very large volumes (∼cm^3^) with good enough contrast and resolution (∼1 *μ*m) to be used for neuroanatomical studies on the whole mouse brain [7]. Since a typical field of view of the objective is ≈400×400*μm*^2^ and dimensions of each raw image may be 512×512 pixels, at least 25×25 tiles are needed to cover the whole volume, each composed by 10.000 slices. Actually, the number of tiles is substantially higher, since acquiring overlapped tiles is necessary to enable automatic and very precise stitching. This increases the dimension of the stitching problem of at least two orders of magnitude, leading to acquisitions of hundreds of large tiles and final image sizes ranging from hundreds of gigavoxels to one teravoxel and more.

### Characteristics of teravoxel-sized datasets

To make acquisitions of such large images viable, computer controlled micropositioning stages must be used to acquire tiles arranged according to a bidimensional grid. Tiles physical coordinates can therefore be recorded during acquisition. Due to the range of movements in each dimension (∼cm), even very small mechanical drifts might make these coordinates not precise enough to enable direct reconstruction of the image. Nevertheless, they can definitely allow a precise control over the overlap between adjacent tiles and the *a posteriori* determination of the Region Of Interest (ROI) for each tile where the overlap occurs.

With respect to the image content there are two problems that are relevant to stitching. First, images may contain thin structures that are just larger than the resolution of the microscope, which makes mandatory to perform the stitching with high precision to avoid artifacts due to misalignments of a few pixels. Second, since specimens can be selectively labeled with fluorescent markers, regions with very limited or none information content may occur in the acquired volume. Since these regions do not contain useful alignment information, the stitching algorithm should be capable to deal with them.

### Current software solutions

Different solutions and tools are available for automatic 3D Stitching of large images [1-3]. Their common strategy is the following: (i) performing a pairwise registration through a combination of Phase Correlation (PC) and Normalized Cross-Correlation (NCC), both of which provide an image similarity score, but with different computational requirements and performance, so that NCC is generally used to refine the PC results; (ii) finding a globally optimal placement of tiles using similarity scores; (iii) combining all tiles in a larger image, while correcting lighting differences in the overlapping regions.

Existing 3D-Stitching tools do not seem adequate to address the Stitching of teravoxel-sized datasets because they were designed under the following major assumptions, which are related to the common characteristics of modern high-resolution imaging techniques such as Confocal, Bright field or Electron microscopes. First, the specimen can be very large along the two radial directions only, whereas the extension along the scanning direction is much more limited. This implies that there are at most hundreds of slices per tile instead of thousands. Second, the number of tiles is limited and their spatial placement can be either organized (i.e. prior knowledge of tiles coordinates is available and at least partially reliable) or un-organized (i.e. prior knowledge of tiles coordinates is missing or unreliable). Note that in the latter case, step (i) must be performed for all possible pairs of tiles making the task computationally tractable only if the number of tiles is limited. Finally, as a consequence of previous assumptions, the overall size of datasets can vary from hundreds of megavoxels up to a few gigavoxels.

For these reasons, the typical stitching tool today addresses some, but not all of the aspects of teravoxel-sized datasets.

### Software design considerations and requirements

Moving from the considerations discussed so far we give the following general requirements for a stitiching tool capable to deal with teravoxels-sized images.

### (I) Stitching processing pipeline

Similarly to other state-of-the-art tools, the stitching process has to perform the following main steps: (i) find the relative position between each pair of adjacent stacks (in fact, since in our case tiles are parallel along the axis direction, there are no rotations, and this simply means to find the displacement between them); (ii) find a globally optimal placement of stacks in the final image space; (iii) combine all stacks into a single 3D image and substitute overlapping regions with a blended version of them.

To effectively deal with the huge dimensions of this class of images one more step to be added is the saving of the resulting 3D image into a multiresolution representation suited for efficient image retrieval and processing.

### (II) Minimization of the overall computational workload

The computational complexity and memory requirements of the algorithms that process tiles should be minimized, while at the same time multiple I/O operations on the same data should be avoided. Since dataset dimensions is expected to further grow as long as microscope techniques develop their potentials, all algorithms, including for what is concerned with I/O operations, should also be parallelizable to make the whole process scalable.

### (III) Identification of indices measuring the alignment quality

Since there may be portions of the volume with scarce or none information content, some of the computed displacements at step (i) may be wrong. This produces a redundant set of alignment data of different quality, which should be associated to some quality index and then globally composed minimizing a measure of the overall error.

### (IV) Manual intervention

When dealing with images with hundreds of tiles, a few wrong computed alignments are likely to occur since thresholds used by the algorithms to deal with alignment quality indices may fail in some cases. In these cases, even if only very few stacks were incorrectly stitched, the entire stitching pipeline should be repeated from the beginning with different settings or the introduced artifacts accepted. In order to avoid this situation, the user should be able to intervene manually by easily detecting and possibly correcting the abnormalities before the final image is produced. To make this possible, stitching steps should be separable thanks to a loading/saving feature for metadata describing their results, which the user should easily edit. A preview-like feature should also be provided to check the effectiveness of the manual intervention on a limited portion of the acquired volume, without processing again all data from scratch.

### (V) Independence of sophisticated content-based analysis of the images

The stitching process should not depend on sophisticated content-based analysis of the raw images, which is likely to generate too much computational workload on very large datasets. In practice, all algorithms should depend at most linearly on the dataset size. Analyses requiring more than linear algorithms, if any, should be performed on limited regions of the acquired volume, and only after a nonredundant, easy to access representation of the whole 3D image has been generated.

### (VI) Expandability

Given the wide variety of images in microscopy and the concrete possibility that new techniques will be soon developed to acquire teravoxel-sized datasets, it may be expected that modified, customized or totally new algorithms will be needed in some of all steps of the stitching process. Hence, it is essential that the software structure of the stitching tool provides means for incorporating new capabilities.

## Implementation

### Architecture

**Figure 1:**
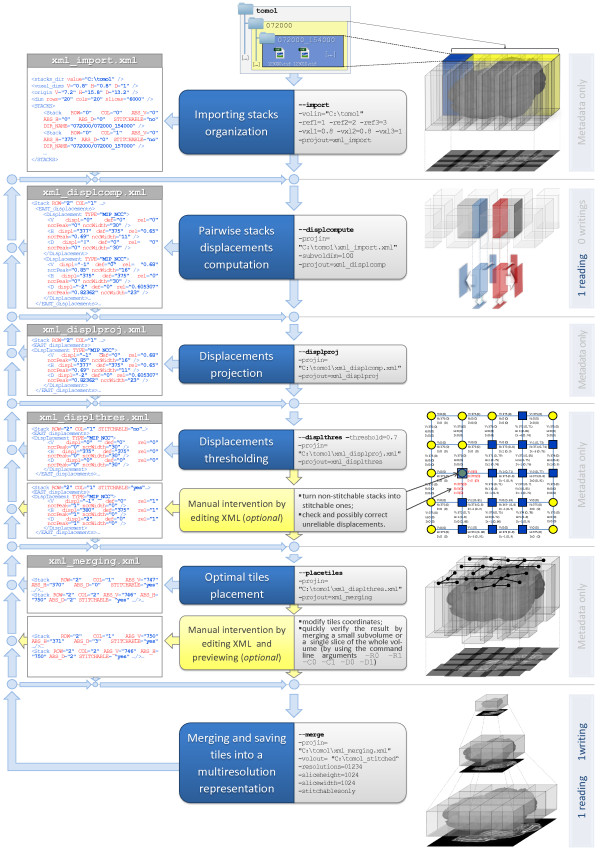
**The stitching pipeline.** The central column lists both automatic and optional manual steps that compose the pipeline. For each automatic step are shown alongside the corresponding command line arguments as well as their values used in a sample workflow. The left column shows the metadata flow and the most significant portions of XML files for each step. On the right column there are some figures illustrating the elaboration of both data and metadata and the number of data readings and writings. The figure which illustrates the manual refinement after the *Displacements thresholding* step was obtained by using the MATLAB scripts we provide for a more comprehensive analysis of metadata. Stitchable stacks are marked in blue (dark grey) and non stitchable ones are marked in yellow (light grey).

As reported in Figure 1, the TeraStitcher tool is structured in a stepwise fashion according to requirement (I). First, a preliminary step that imports data organization is necessary to access data properly and efficiently in subsequent steps. We assume raw data arranged as a tile matrix whose dimensions will be referred to as *vertical* (*V*) and *horizontal* (*H*). Data are stored according to a two-level hierarchical structure where, at the first level, directories contain all tiles of a row or of a column and at the second level directories contain the tile corresponding to that row or column, stored as a sequence of slice images disposed along the *depth* (*D* in the following) direction. This allows us to load just a small portion of data at the time, which is a necessary condition for requirement (II). Note also that directory names and image files names convey the information about tile and slice positions as provided by the controlling software of the microscope, so that this information can be extracted in the import step and used whenever required. Second, the *Pairwise stacks displacements computation* step produces a redundant set of alignment data with associated reliability measures, according to requirement (III). Both redundancy and reliability measures are exploited to select the most reliable displacement for each pair of adjacent stacks (*Displacements projection*), to discard the unreliable displacements (*Displacements thresholding*) and to find an optimal placement of tiles (*Optimal tiles placement*) before merging tiles into a multiresolution representation. The details of each step are discussed in section Algorithms. Third, most steps produce metadata describing their results and/or use these metadata to perform their tasks. At each step, metadata are saved in XML files which the user can edit to manually refine displacements, tile coordinates or other stitching metadata, according to requirement (IV). The details of this approach are given in section Manual intervention.

**Figure 2:**
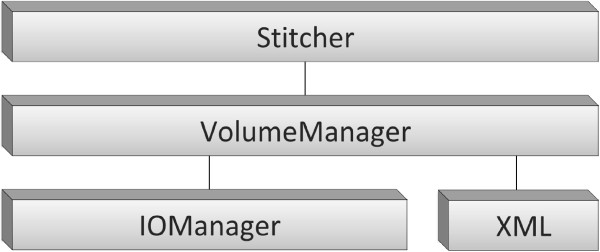
**The software architecture of TeraStitcher.** The architecture of the proposed TeraStitcher tool is composed by four modules organized in three layers with growing abstraction levels. At the lowest abstraction level, the *IOManager* and *XML* modules contain input/output routines for data and metadata, respectively. The middle layer contains the *VolumeManager* module, which is responsible to model data organization and to access both data and metadata through functionalities provided by the lower layer. At the top layer, the *Stitcher* module implements the stitching pipeline.

In order to satisfy the expandability requirement (VI), we followed an object-oriented approach in designing the software architecture of our tool. It consists of four modules organized in three layers with growing abstraction levels (see Figure 2). At the lowest abstraction level, the *IOManager* and *XML* modules contain input/output routines for data and metadata, respectively. The middle layer contains the *VolumeManager* module, which is responsible to model data organization and to access both data and metadata through functionalities provided by the lower layer. At the top layer, the *Stitcher* module implements the stitching pipeline.

**Figure 3:**
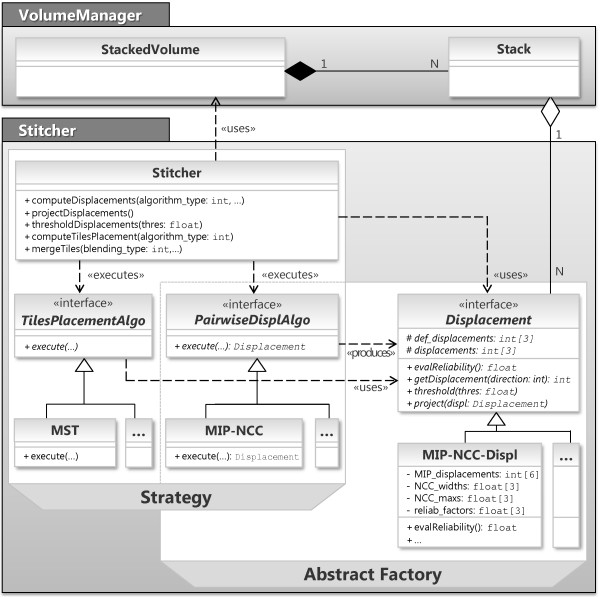
**Class diagrams of *VolumeManager *and *Stitcher *modules.** A *StackedVolume* contains as many *Stack* objects as the number of stacks that compose the volume and it is responsible to model data organization. The *Stitcher* methods implement the stitching pipeline and the strategies to use efficiently system resources, by accessing data through the *StackedVolume*. These strategies use the algorithms *TilesPlacementAlgo* and *PairwiseDisplAlgo* that may depend on the specific characteristics of images. Note that *TilesPlacementAlgo* uses *Displacement*s produced by *PairwiseDisplAlgo*. Classes *MST*, *MIP-NCC*, and *MIP-NCC-Displacement* implement the actual algorithms used in our implementation.

Let us have a look inside the *Stitcher* module, which is critical when considering the expandability and generality requirements of our tool. The internal structure of *Stitcher* is shown in Figure 3. *Stitcher* methods implement the stitching pipeline of Figure 1 and the corresponding strategies to use efficiently system resources. Two design patterns, *Strategy* and *Abstract Factory*[8], are used to create a layer of interfaces separating *Stitcher*’s strategies from the actual algorithms and data structures, respectively, since these may depend on the specific characteristics of the acquired images. *Strategy* lets the user determine at runtime which *PairwiseDisplAlgo* and *TilesPlacementAlgo* algorithms should be used, whereas *Abstract Factory* lets a *PairwiseDisplAlgo* produce its own *Displacement*. A concrete subclass of *Displacement* should provide not only spatial information (i.e. the relative position of adjacent tiles), but also one or more displacement reliability measures tailored to the adopted alignment strategy, with values between 0 (totally unreliable displacement) and 1 (reliable displacement). In addition, its *project*(*displ*) and *threshold*(*thres*) methods should use these reliability measures to implement the *Displacement projection* and *Displacement thresholding* steps respectively, as explained in more detail in section Algorithms. So, new implementations of steps 3-6 of the stitching pipeline can easily be embedded within this framework by implementing at most three class interfaces.

### Algorithms

#### Pairwise stacks displacement computation

**Figure 4:**
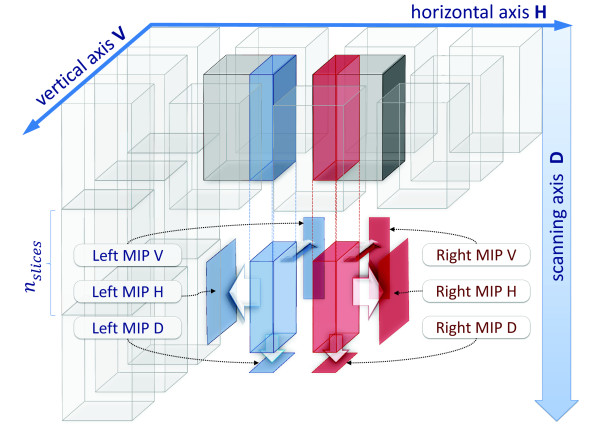
**Multi-MIP-NCC method.** Each tile is split along the *D* direction into substacks of *n*_*slices*_slices. For each couple of adjacent substacks, three MIPs along the three directions are extracted from the overlapping regions. Then a 2D-NCC map is computed for each pair of homologous MIPs, so obtaining two displacements for each direction. Two reliability measures extracted from the NCC map are used to select for each direction the best of the two available displacements.

To compute the relative position of a pair of tiles (*Pairwise stacks displacements computation* step) we adopted a multi-MIP-NCC approach (see Figure 4). Tiles are split along the *D* direction into substacks of *n*_*slices*_ slices, where the parameter *n*_*slices*_ can be set by the user with typical values in the range [ 100,200]. For each couple of homologous substacks of two adjacent tiles, the overlapping regions are identified using tile positions provided by the instrument. The information contained in these regions is condensed in three 2D images computed as maximum intensity projections (MIPs) along the three dimensions. Then a 2D-NCC map is computed for each of the three pairs of MIPs in a search region of side 2*δ*_*search*_ + 1 pixels centered on the central pixel of the overlapping regions. In this way, two displacements corresponding to NCC peaks are obtained for each direction, for a total of 6 displacements. The most reliable displacement for each direction is then selected and memorized using a combination of two reliability measures extracted from the NCC maps. These measures are the NCC peak value (the higher, the more reliable) and the NCC shape width of the peak (the smaller, the more reliable). The informations collected so far are stored in a *MIP-NCC-Displ* object (see Figure 3). Note that pairs of substacks are aligned separately, producing a redundant set of alignment data for each pair of adjacent tiles. Such a redundancy is exploited in the *Displacement projection* step, as explained below.

Besides the relevant reduction in memory occupancy memory requirements reduction, which is discussed in section Strategies to limit resource requirements, there are two reasons for dividing tiles into substacks. First, since multi-MIP-NCC is based on MIP projections, it works well if the information content of the overlapping regions in not too dense. Hence, it could perform poorly if applied to whole stacks, whose MIPs along the *D* direction could accumulate too much information. Second, working on a few relatively small 2D matrices at the time allows an efficient use of the memory hierarchy. The only minor drawback is a small increase in computational workload, since multiple NCC maps have to be computed along *D*.

#### Displacement projection

Once all substacks of one tile have been aligned with their adjacent homologous, the obtained displacements are combined to give the best displacement for each pair of adjacent tiles. Such a combination is implemented in the *project*(*..*) method of *MIP-NCC-Displ* class and it is done by selecting, for each direction separately, the most reliable single direction displacement. This is possible because each single direction displacement has its own reliability measures, thanks to the adopted *MIP-NCC* approach. Note that, even if independent reliability measures were not available for each direction, one could still select the most reliable displacement among the ones computed for every substack. In any case, multiple substacks alignments provide an advantage, since substacks difficult to stitch are likely to occur.

It is worth noting that having multiple substacks alignments could also be used to cope with situations where different reliable alignments in *V* and *H* directions are returned by the MIP-NCC algorithm. This would correspond to acquisitions where sample movements in *D* direction where not perfectly parallel and tiles should be someway rearranged before merging. While on the one hand this would require tile resampling with a remarkable increase in computational costs, on the other hand we did not actually observe such shifts along the *D* direction in our acquisitions. For this reasons the tool uses just one displacement (the best in every direction) to align adjacent tiles.

#### Displacement thresholding

After the best displacements have been obtained, their reliability is thresholded: when it is below the given threshold, the default displacement provided by stage coordinates is reestablished and the corresponding reliability measure is set to 0 (totally unreliable). Furthermore, the associated pair of stacks is marked as a *nonstitchable* pair. If all the four pairs associated with a tile are nonstitchable, then the tile itself is marked as nonstitchable, i.e. there’s no way to stitch it to any other stacks, except of using the tile position estimation provided by the instrument. This information is taken into account in the next step.

We observed from different datasets that good candidate values for displacement threshold are in the range [ 0.7,0.8], as also suggested by the results reported in Table 1. However, we do not exclude that in the case of low-contrasted or high noisy data these values could be lower.

**Table 1:** Comparison of displacements errors and quality measures among different datasets

**Dataset**	**Tool**	**Error**						**Quality measure**					
		**Max**			**Average**			**Min**			**Average**		
		**V**	**H**	**D**	**V**	**H**	**D**	**V**	**H**	**D**	**V**	**H**	**D**
	TeraStitcher	0	0	0	0	0	0	0.86	0.85	0.86	0.87	0.87	0.88
	Fiji	0	0	0	0	0	0		0.51			0.63	
C. elegans worm^a^	XuvStitch	0	0	0	0	0	0		0.72			0.78	
	iStitch	0	0	0	0	0	0		0.94			0.95	
	TeraStitcher	0	0	0	0	0	0	0.79	0.80	0.64	0.81	0.83	0.73
	Fiji	0	0	0	0	0	0		0.55			0.58	
Neuron filaments^a^	XuvStitch	0	0	0	0	0	0		0.67			0.76	
	iStitch	0	0	1	0	0	0.3		0.90			0.92	
	TeraStitcher	0	0	0	0	0	0	0.81	0.88	0.75	0.88	0.92	0.76
	Fiji	0	0	0	0	0	0		1			1	
Fruit fly brain^b^	XuvStitch	0	0	0	0	0	0		1			1	
	iStitch	0	0	0	0	0	0		0.95			0.96	
	TeraStitcher	1	0	1	0.5	0	0.5	0.71	0.68	0.53	0.73	0.7	0.61
Portion of mouse cerebellum^c^ (axons)	Fiji	0	0	0	0	0	0		0.72			0.73	
	XuvStitch	0	0	0	0	0	0		0.2			0.31	
	iStitch	7	1	0	3.5	0.5	0		0.95			0.96	
	TeraStitcher	0	0	0	0	0	0	0.84	0.85	0.87	0.87	0.87	0.89
Portion of mouse brain^c^ (neurons and axons)	Fiji	0	0	0	0	0	0		0.77			0.80	
	XuvStitch	0	0	0	0	0	0		0.90			0.91	
	iStitch	w.a.^d^	w.a.	w.a.	w.a.	w.a.	w.a.		0.76			0.78	
	TeraStitcher	0	0	0	0	0	0	0.76	0.77	0.6	0.8	0.84	0.75
Whole mouse cerebellum^c^ (Purkinje cells and axons)	Fiji	0	0	0	0	0	0		0.78			0.85	
	XuvStitch	3	3	2	0.8	0.8	1		0.75			0.87	
	iStitch	1	0	0	0.2	0	0		0.92			0.94	
	TeraStitcher	0	0	0	0	0	0	0.77	0.75	0.74	0.77	0.77	0.75
Whole mouse brain^c^ (neurons and axons)	Fiji	0	0	0	0	0	0		0.37			0.39	
	XuvStitch	0	0	0	0	0	0		0.6			0.65	
	iStitch	0	0	0	0	0	0		0.77			0.79	

In this table we report a comparison of displacement errors (in voxels) and quality measures between TeraStitcher and the state-of-the art stitching tools when stitching Megavoxel-sized datasets.

^a^Publicly available dataset downloaded from http://www.vaa3d.org.

^b^Publicly available dataset downloaded from http://www.xuvtools.org.

^c^CLSM dataset (courtesy of F.S. Pavone, L. Sacconi, L. Silvestri) available at http://www.iconfoundation.net.

^d^Wrong alignment.

#### Optimal tiles placement

**Figure 5:**
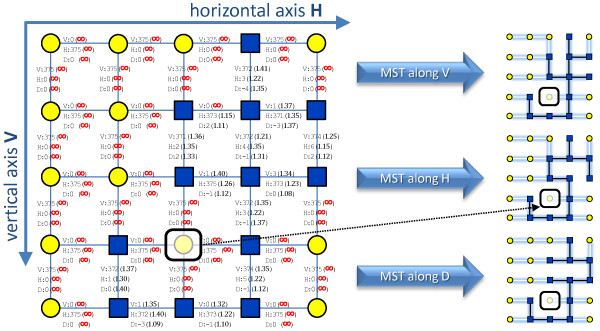
**Optimal tiles placement using a minimum spanning tree (MST).** Weighted undirected graph of tiles whose edges represent displacements. Weights are computed as the inverse of displacements reliability measures, yielding + *∞*for the unreliable ones. These prevents the tree connecting stitchable tiles (marked as blue squares) from passing through the nontistchable ones (marked as yellow circles). If different reliability measures are available along the three directions, as in the case of using MIP-NCC to compute misalignments, three minimum spanning trees are obtained for each direction separately. The marked tile in the figure is a nonstitchable tile which is excluded from any MST path traversing stitchable tiles.

Since displacements provided by previous steps are not independent, a globally optimal placement of tiles has to be found before producing the final representation of the whole 3D image. Following [3], we computed the minimum spanning tree of the undirected weighted graph constituted by the mesh of tiles connected by edges representing tiles displacements. The minimum spanning tree algorithm finds the edges (i.e. the displacements) connecting all tiles without forming cycles and whose total weight is minimum. Weights are computed as the inverse of the displacements reliability measures (see Figure 5). In this way, displacements associated to nonstitchable stacks have a weight equal to + *∞* since their reliability was set to 0. This prevents the tree connecting stitchable stacks from passing through nonstitchable ones, unless there is a group of isolated stitchable stacks, in which cases a path including them is provided anyway. If different reliability measures are available along the three directions, as in our case after using MIP-NCC to compute misalignments, the algorithm is applied separately for each direction to find the alignments that are globally optimal for that direction.

#### Merging and multiresolution generation

After all relative displacements among tiles have been computed, tile merging is performed.

**Figure 6:**
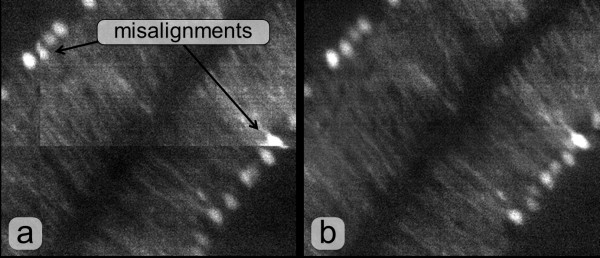
**Result of stitching at the border of four overlapping tiles.** Merging without aligning nor blending **(a)** and with aligning and blending **(b)**.

Final slices are produced simply copying nonoverlapping regions, while the overlapping ones are substituted by a blended version of them. Blending is performed using two sinusoidal functions phase-shifted by *Π* for weighting the pixels of the two images (see Figure 6-b).

Finally merged slices are saved at several resolutions. At every lower resolution the downsampling is done by computing the mean of an 8-pixels cube of the higher resolution. The resulting image can be saved either as a single stack or in a multistack format with the dimensions of individual stacks decided by the user. The multistack format is provided since it allow a selective access to the higher resolution image, which dramatically improves memory efficiency of further processing.

All these operations are performed according to a strategy minimizing memory occupancy, which is described in detail in the next section.

### Strategies to limit resource requirements

In this section we discuss how the entire stitching procedure is organized to minimize both I/O data transfers and memory requirements which are critical problems in processing data of TB size. We will focus on the two stitching steps that process data, which are the *Pairwise stacks displacements computation* and the *Merging and saving tiles into a multiresolution representation* steps. As reported in Figure 1, data are read once for each of the two steps. To understand why, one should note that the final representation of the whole 3D image cannot be produced if the precise relative positions of tiles have not been determined. Tile positioning in turn requires that all tile pairs have been properly aligned. Since the entire dataset cannot be kept in memory, all data have to be read at least twice and written back to mass storage once, after the final image representation has been produced.

Let us refer to *N*_*slices*_ as the number of slices per tile and to *N*_*rows*_and *N*_*cols*_ as the number of rows and columns of the tile matrix, respectively. Each tile which is not on the matrix border has four adjacent tiles, conventionally referred to as *West*, *North*, *East* and *South*, respectively.

**Figure 7:**
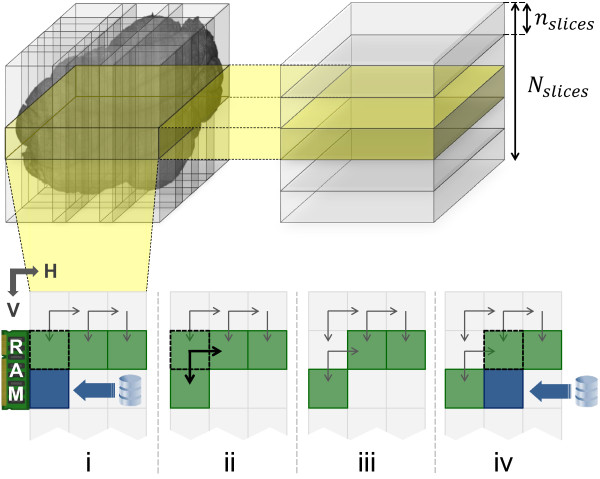
**Memory management in the pairwise stacks displacements computation step.** The whole volume is processed a layer at the time, each composed by *n*_*slices*_slices. For each layer, stacks are read row-wise when there are more rows than columns (i-iv). A new substack is loaded **(i)** when its *North* substack needs it for displacement computation **(ii)**. After that, the *North* substack can be released **(iii)** and this process is repeated for the next column **(iv)**.

As mentioned in section Algorithms, in the *Pairwise stacks displacements computation* step the whole volume is processed a layer at the time, each composed by *n*_*slices*_slices at most. For each layer, tiles are read row-wise or column-wise depending on which dimension is smaller. Supposing that stacks are read row-wise, a stack is maintained in memory until its displacements with its *South* and *East* stacks are computed, after which it is released (see Figure 7). If border tiles are properly managed skipping alignments with missing adjacent tiles, only *N*_*cols*_ + 1 tiles have to be maintained in memory to compute all pairwise displacements, and all data have to be read only once. The column-wise case is symmetric. Hence, this step needs to keep in memory only (min{*N*_*rows*_,*N*_*cols*_} + 1)×*n*_*slices*_slices at the time. Such a quantity can be directly controlled by the user by choosing a small value for *n*_*slices*_.

**Figure 8:**
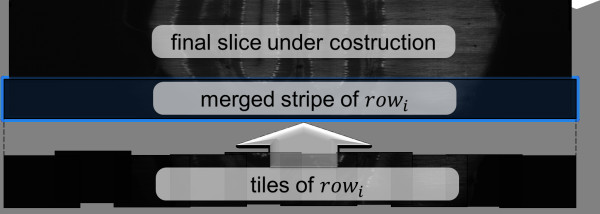
**Memory management in the merging step.** The whole volume is processed a layer at the time, each composed by the number of slices needed to generate the lowest resolution (e.g. 32 slices for a reduction of 5 times). For each layer, stacks are read row-wise and merging is performed slice by slice. When one slice group of every tile in row *i* of the tile matrix have been read, adjacent tiles in the groups are merged using the blending algorithm described in the Algorithms section. This produces a horizontal stripe, which is merged with the ones previously generated from the slices of the preceding rows, so producing larger stripes until the whole slice of the final image representation is obtained.

In the *Merging* step, only a small group of slices for each tile are read at the time, following again a row-wise order for tiles, and taking into account for each tile the right displacement along *D* computed after the first step. When one slice group of every tile in row *i* of the tile matrix have been read, adjacent slices in the groups are merged with the algorithm described in section *Algorithms*. This produces a group of horizontal stripes of the slices of the final image representation. These stripes are then merged with the ones previously generated from the slices of the preceding rows, so producing larger stripes of the same group of final slices (Figure 8).

Managing properly the first stripe and repeating this procedure for all rows of the tile grid, a group of complete slices of the final representation is produced and saved back to mass storage. In this way, the memory requirement is to keep in memory only a small number of final slices. The number is determined by the minimal resolution of the whole 3D image that has to be generated and by the downsampling method adopted. Groups of a few tens of slices are enough in practice. Since a resolution reduction of 5 times is enough for efficient access to data, in practice there are 2^5^=32 slices in each group.

We conclude the section observing that in both steps tiles are divided in multiple groups of slices and each group is processed independently. Parallelization of the whole process is therefore trivial and very efficient. Indeed, if groups of slices of all tiles are distributed on multiple disks and multiple processing units are available, all operations can be parallelized with negligible overhead, including I/O operations.

### Manual intervention

In this section we discuss how the tool enables easy and fast manual intervention in order to refine stitching metadata such as pairwise displacements, tiles coordinates and stitchable/nonstitchable attributes of stacks. Our solution is based on three principles: (i) using XML files to load/save stitching metadata, (ii) enabling a preview-like feature to stitch selectable portions of data and (iii) providing graphical models of metadata for a more comprehensive analysis. In the following we detail each of these principles.

#### XML files editing

Stitching steps save their metadata into XML files and they can be launched individually by loading an existing XML containing the metadata used by that step. The benefit of this approach is threefold. First, the user can manually refine stitching metadata by simply editing XML files, which structure was designed to provide easy readability and understanding (see Figure 1). Second, steps become separable not only logically but also physically by saving/loading the state of the stitching process into/from the XML file. It is then easier to tune single-step parameters without re-executing the previous ones. Third, steps dealing only with metadata can be performed on different machines. This enables manual intervention on any machine when the result does not need to be verified on data (e.g. consider the intervention between *Displacement thresholding* and *Optimal tiles placement* step).

#### Preview-like feature

Each step dealing with data lets the user select the portion of volume to be processed in terms of an interval of stacks rows and/or columns of the tile matrix and/or in terms of an interval of slices along *D* axis. The latter is very useful to verify the quality of stitching before and after any manual intervention. Thanks to the adopted slice-based merging strategy, the tool enables fast stitching of small subset of slices of the whole volume, so obtaining a preview of the final volume in just few seconds. After disabling any activated blending method, the user can then easily detect and locate abnormalities on this preview by visual inspection on stacks borders.

#### Graphical models of metadata

We developed and provide MATLAB scripts that starting from XML files generate graphical models of metadata to be used for a more comprehensive analysis and faster manual intervention. An example is given in Figure 1 for the *Displacement thresholding* step, where we report a map of stitchable and non stitchable stacks together with their displacements values and reliability measures. From this map, one could observe if the connected region of stitchable stacks does correspond to the shape of the acquired specimen and if the alignment of some nonempty stacks turned out to be particularly difficult along a particular direction. In this way, the user can detect abnormalities directly on metadata and then edit the corresponding XML entry.

## Results and discussion

In this section we provide four sets of performance data characterizing our stitching tool from both qualitative and quantitative points of view. A comparison between the TeraStitcher tool and the ones presented in [1-3] is also provided in both cases.

First, in Table 1 we report maximum and average displacements errors as well as their corresponding quality measures obtained by stitching tiles from different datasets with our tool and the ones it has to be compared with. The aim of such a comparison is to demonstrate that TeraStitcher performs at least as well as the others from a qualitative point of view. To this purpose, several image stacks from 7 datasets differing in content, bitdepth, lighting conditions, contrast and SNR have been used. Displacements errors were measured taking into account an uncertainty of ±1 voxel of the groundtruth, which was then subtracted from each error. Quality measures were normalized from [ 0,100] to [ 0,1] for *XuvStitch*. The results show that TeraStitcher can stitch both images acquired in CLSM and images acquired with other microscopy techniques with single-pixel precision. Conversely, both *iStitch* and *XuvStitch* didn’t perform well with CLSM images. In fact, the former provided more than one wrong displacement. However, differently from other tools considered here, this tool does not use stage coordinates, which probably made stitching more difficult. As to *XuvStitch*, it provided just wrong displacements in two instances. Moreover, its quality measures were quite low in all the instances of the *Mouse cerebellum* dataset, which would make them not suited to be used as reliability measures. Finally, *Fiji* performed very well in all the instances considered, but its quality measures seemed to be content-dependent and they were very low for 3 of the 7 considered datasets.

Second, in Table 2 we report a comparison about the execution times and memory occupancy of individual phases as well as of the whole stitching procedure on megavoxel-sized data extracted from the *Whole mouse cerebellum* dataset acquired in CLSM. Measurements have been carried out on a laptop equipped with 4 GB of RAM, 500 GB of disk space, 1 dual-core CPU at 2.53 GHz. We used the following values for TeraStitcher parameters: *n*_*slices*_=100, *δ*_*search*_=16. In order to have a fair comparison, the other tools parameters having the same meaning of *δ*_*search*_ were set at the same value. It is worth noting that although *iStitch* implementation does not exploit the knowledge of the approximate relative position of the two stacks as the other tools considered do, it computes an initial relative position on a quite undersampled image (see [3] for details). For this reason the contribution of this preliminary step to the overall execution time can be considered negligible. The results show that both execution times and memory occupancy of our tool are significantly lower than those of the others, except for *XuvStitch*, whose execution times were comparable with those of TeraStitcher. However, its memory peak becomes significantly higher than that of TeraStitcher when stitching large datasets, as it is shown in the third row of the table, where four stacks of 512×512×600 voxels have been stitched. Another interesting result is that *iStitch* performed better than the other tools when stage coordinates are not provided, probably thanks to the preliminary step on the undersampled image mentioned above, which confirms to be quite effective.

**Table 2:** Performance comparison among stitching tools

**Data size (MVoxel)**	**Stacks**	**Tool**	**Use of stage coordinates**	**Memory peak**	***T***_***stitching***_^**a**^	***T***_***displ. comp.***_	***T***_***merging***_	***T***_***I*/*O***_	*T*_*total*_
		TeraStitcher	yes	200	4	2	2	7	11
		Fiji	yes	450	45	20	25	8	53
		Fiji	no	1400	99	75	24	3	102
100	2×200 slices	XuvStitch	yes	400	n.a.	4	16	n.a.	20
		XuvStitch	no	950	n.a.	22	16	n.a.	38
		iStitch	no	600	11	n.a.	n.a.	n.a.	17
		TeraStitcher	yes	300	8	4	4	12	20
		Fiji	yes	1300	132	62	71	13	145
200	4×200 slices	XuvStitch	yes	500	n.a.	6	28	n.a.	34
		iStitch	no	662	37	n.a.	n.a.	n.a.	46
		TeraStitcher	yes	300	35	23	12	32	20
		Fiji	yes	> 3000	n.a.	113	n.a.	n.a.	n.a.
600	4×600 slices	XuvStitch	yes	1200	n.a.	18	80	n.a.	34
		iStitch	no	1900	143	n.a.	n.a.	n.a.	46

In this table we report a comparison of execution times (in seconds) and memory peaks (in MB) between TeraStitcher and the state-of-the art stitching tools when stitching Megavoxel-sized datasets.

^a^Time spent in stitching, net of I/O.

Third, in Table 3 we report execution times and memory peaks of individual phases when stitching gigavoxel and teravoxel-sized data with TeraStitcher. We could not make a comparison with the other tools because they easily ran out of memory with such data. Measurements have been carried out on a workstation equipped with 96 GB of RAM, 9 TB of disk space, 2 quad-core CPUs at 2.26 GHz and using the following values for TeraStitcher parameters: *n*_*slices*_=100, *δ*_*search*_=25. The first three rows refer to increasing subvolumes of a 3D image of the *Whole mouse cerebellum* dataset. It is apparent that all times are linearly dependent on the image size. The complete image (∼200 gigavoxels) has been processed in about 15 hours (third row in the table). The last row refers to the time needed to process a 1.3 teravoxels image corresponding to about 4 days. Times corresponding to individual substeps are not available in this case.

**Table 3:** Performances of TeraStitcher when stitching very large datasets

**Data size (Gigavoxel)**	**Dataset**	**Memory peak**	***T***_***stitching***_	***T***_***displ. comp.***_	***T***_***merging***_	***T***_***I*/*O***_	***T***_***total***_
63,25	Portion of whole mouse cerebellum	821	60	23	37	157	217
126,50	Portion of whole mouse cerebellum	1132	145	68	77	318	433
198,78	Whole mouse cerebellum	1132	232	109	123	539	771
1315,04	Whole mouse brain	2450	n.a.^a^	n.a.	n.a.	n.a.	6050

In this table are shown both execution times (in minutes) and memory peaks (in MB) of the TeraStitcher tool when stitching Gigavoxel and Teravoxel-sized datasets.

^a^Not available.

Finally, we show in Figure 8 the result of the stitching process on slices at the border of four overlapping tiles for different datasets. This example is representative of the very good qualitative performance attained by our alignment and blending algorithms.

## Conclusions

Forthcoming microscopy techniques like Confocal Light Sheet Microscopy are able to acquire tiled images of 1 teravoxel or more. These images pose novel requirements to stitching that are not adequately addressed by existing tools.

In this paper we have presented a tool designed for automatic 3D stitching of teravoxel-sized tiled images. The tool, initially developed for stitching images generated by the CLSM microscope, is completely general, and suited to be adapted to other instruments capable to acquire images of these dimensions. The central idea is to accurately specify the requirements of the stitching problem when teravoxel-sized images are involved, and then use efficiently the system resources to perform the stitching of these images. To improve the generality of the proposed tool, we defined a software architecture that clearly separates the strategies in common among different datasets from the algorithms that may depend on specific characteristics of the acquired images.

An implementation of the tool that is capable to perform the stitching of real teravoxel-sized images on workstations with relatively limited memory resources in reasonable time has been presented. It uses specific algorithms well suited for a relatively wide class of images, that substantially reduce memory and computational requirements of some basic steps of the stitching procedure with respect to previous approaches. A comparison with state-of-the-art stitching tools confirms that the solutions adopted are best suited for stitching very large, automatically acquired microscopy images.

## Availability and requirements

We provide TeraStitcher both as standalone application and as plugin of the free software Vaa3D [9]. Sources and binaries, as well as some MATLAB scripts for the generation of graphical models for metadata, are freely available at the project home page. An online help is also provided which comprises the detailed description of command line parameters, stitching pipeline, supported data formats, supported stacks organization, and a FAQ section. 

● **Project name**: TeraStitcher

● **Project home page**: http://code.google.com/p/terastitcher

● **Operating system**: Platform independent

● **Programming language**: C++

● **Other requirements**: OpenCV library 2.2.0 or higher is required for compiling both the standalone application and the plugin of Vaa3D. On the contrary, our binary packages can be directly used since we provide OpenCV precompiled binaries within them.

● **License**: both source code and binaries are freely available at the project home page for non-commercial purposes only and we require that our work is cited in user’s related studies and publications, if any. A short license agreement which encloses this clause is provided in the header of every source file as well as in the binary packages and before downloading any material related to the project.

## Abbreviations

LSM: Light Sheet Microscopy; LENS: European Laboratory for Non-linear Spectroscopy; CLSM: Confocal Light Sheet Microscopy; ROI: Region Of Interest; PC: Phase Correlation; NCC: Normalized Cross-Correlation; I/O: Input/Output; V: vertical; H: horizontal; D: depth; GB: GigaByte; TB: TeraByte.

## Competing interests

The authors declare that they have no competing interests.

## Authors’ contributions

Both authors worked to the design, development and testing of the TeraStitcher tool. They also equally contributed to write the manuscript. Both authors read and approved the final manuscript.

## References

[B1] Preibisch S Saalfeld S Tomancak P Globally optimal stitching of tiled 3D microscopic image Bioinformatics 2009 25 11 1463 1465 10.1093/bioinformatics/btp184 19346324 PMC2682522

[B2] Emmenlauer M Ronneberger O Ponti A Schwarb P Griffa A Filippi A Nitschke R Driever W Burkhardt H XuvTools: free, fast and reliable stitching of large 3D datasets J Micros 2009 233 42 60 10.1111/j.1365-2818.2008.03094.x 19196411

[B3] Yu Y Peng H Automated high speed stitching of large 3D microscopic images Proceedings of IEEE 2011 International Symposium on Biomedical Imaging: From Nano to Macro: March 30-april 2 2011 Chicago 238 241

[B4] Keller P Dodt H Light sheet microscopy of living or cleared specimens Curr Opin Neurobiology 2012 222 138 143 10.1016/j.conb.2011.08.003 21925871

[B5] Verveer P Swoger J Pampaloni F Greger K Marcello M Stelzer E High-resolution three dimensional imaging of large specimens with light sheet based microscopy Nat Methods 2007 4 311 313 17339847 10.1038/nmeth1017

[B6] Dodt H Leischner U Schierloh A Jährling N Mauch C Ultramicroscopy: three-dimensional visualization of neuronal networks in the whole mouse brain Nat Methods 2007 4 331 336 10.1038/nmeth1036 17384643

[B7] Silvestri L Bria A Sacconi L Iannello G Pavone F Confocal light sheet microscopy: micron-scale neuroanatomy of the entire mouse brain Optic Express 2012 20 18 20582 20598 10.1364/OE.20.020582 23037106

[B8] Gamma E Helm R Johnson R Vlissides J Elements of Reusable Object-Oriented Software 1995 Addison-Wesley Boston: Longman Publishing Co.

[B9] Peng H Ruan Z Long F Simpson J Myers E V3D enables real-time 3D visualization and quantitative analysis of large-scale biological image data sets Nat Biotechnol 2010 28 4 348 353 10.1038/nbt.1612 20231818 PMC2857929

